# Gamma-Delta Hepatosplenic T-Cell Lymphoma in a Two-Year-Old: A Case Report

**DOI:** 10.7759/cureus.102138

**Published:** 2026-01-23

**Authors:** Hibah Mohammed, Adib Khan, Fiona Lin, Amia Mourad, Madhura Butala

**Affiliations:** 1 Medicine, Lake Erie College of Osteopathic Medicine, Bradenton, USA; 2 Pediatrics, Ascension St. Vincents, Jacksonville, USA

**Keywords:** gamma-delta t-cell, hepato-splenic lymphoma, immunophenotyping, minimal residual disease, pediatric cancer, t-cell lymphoma

## Abstract

Gamma-delta (γδ) hepatosplenic T-cell lymphoma (HSTCL) is a rare, aggressive malignancy that is exceptionally uncommon in young children. It often presents with nonspecific symptoms that delay diagnosis. We describe a previously healthy 26-month-old girl who developed daily fevers, weight loss, decreased appetite, and progressive lower-extremity weakness leading to loss of ambulation. Examination revealed marked hepatosplenomegaly without lymphadenopathy, and laboratory studies showed lymphocytic leukocytosis with evolving cytopenias. Bone marrow evaluation identified a clonal γδ T-cell population, and liver biopsy confirmed sinusoidal infiltration, establishing the diagnosis of γδ HSTCL. Her clinical course was characterized by poor response to multiple induction, consolidation, and targeted therapies. She presented with persistent minimal residual disease (MRD), requiring repeated treatment escalation and substantial supportive care. This case illustrates the diagnostic difficulty and therapeutic resistance of pediatric γδ HSTCL. Early recognition, molecular characterization, and timely consideration of possible stem cell transplant are essential to improving outcomes in this rare and highly aggressive pediatric lymphoma.

## Introduction

T-cells are classified into two main lineages based on the composition of their T-cell receptors (TCRs): alpha-beta (αβ) T-cells and gamma-delta (γδ) T-cells. In human peripheral blood, αβ T-cells constitute approximately 65-75% of mononuclear cells, while γδ T-cells account for less than 10% of mononuclear cells [1]. Neoplasms derived from γδ T-cells represent a rare and biologically distinct group of lymphoid malignancies [2]. Under the current World Health Organization/International Consensus Classification (WHO/ICC), neoplasms derived from γδ T-cells include distinct clinicopathologic entities such as hepatosplenic T-cell lymphoma (HSTCL) and primary cutaneous γδ T-cell lymphoma [3].

γδ HSTCL is challenging to diagnose due to its rarity and the absence of lymphadenopathy. Patients often present with constitutional symptoms and generalized abdominal discomfort, which can mimic acute leukemia or infectious processes. Cytopenias, particularly thrombocytopenia, are common and correlate with disease severity [4]. Unlike many other lymphomas, nodal involvement is rare. Instead, γδ HSTCL primarily presents with sinusoidal infiltration of the liver, spleen, and bone marrow by neoplastic γδ T cells, leading to hepatosplenomegaly. This makes histopathology and immunophenotyping essential for diagnosis [5]. The neoplastic cells are cytotoxic T-cells with γδ TCRs, typically showing the following phenotype: CD2+, CD3+, CD4-, CD5-, CD8-/+, CD56+/- [4]. A small subset expresses αβ TCR, which is described as a variant of HSTCL. The tumor cells have a nonactivated cytotoxic T-cell immunophenotype and frequently carry recurrent cytogenetic abnormalities. Characteristic genetic features of HSTCL include isochromosome 7q, trisomy 8, and activating mutations of JAK/STAT pathway genes (*STAT5B*, *STAT3*) and chromatin-modifying genes (*SETD2*, *INO80*, *ARID1B*) [4]. These molecular features may represent potential therapeutic targets. 

γδ HSTCL demonstrates a strong male predominance; the median age of diagnosis is 34 years [4]. Given its aggressive course and poor prognosis, early detection is critical. There are very few documented pediatric cases of γδ HSTCL, which often results in delayed diagnosis and initiation of treatment.

Therapeutic management of pediatric HSTCL remains exceptionally challenging due to its pronounced chemoresistance and aggressive clinical course. Conventional multi-agent chemotherapy regimens, including anthracycline-based protocols, often achieve only transient responses, with the majority of patients experiencing early relapse and poor long-term survival [6]. Across both pediatric and adult populations, median overall survival is typically less than 18 months, and sustained remission with chemotherapy alone is uncommon [6]. 

We report a case of γδ HSTCL in a toddler, contributing to the limited pediatric literature and emphasizing the diagnostic and therapeutic challenges of this aggressive disease in very young patients.

## Case presentation

An 18-month-old previously healthy female, at the 92nd weight-for-length percentile, initially presented to the gastroenterology clinic with constipation and occasional bloody stools. At that time, she had no weight loss, abdominal pain, distension, or organomegaly. She was started on lactulose, which provided minimal relief, and she continued to experience recurrent constipation over the subsequent months. At 26 months, she presented to the Emergency Department (ED) with a one-month history of daily fevers reaching 103°F, progressive weight loss, lower-extremity weakness, and right knee pain. She had been unable to ambulate for three days prior to admission. Earlier in the month, she had sought care at multiple outside EDs for persistent fevers; however, no laboratory studies had been obtained during those encounters. The diagnostic delay appeared multifactorial, reflecting both the nonspecific early presentation of this rare malignancy in early childhood and the absence of initial laboratory evaluation during prior ED encounters.

During this ED presentation, a comprehensive laboratory workup was performed. Complete blood count (CBC) revealed an elevated white blood cell (WBC) count of 46.69k/µL with a lymphocytic predominance of 83% (Table 1). Inflammatory markers were elevated, including a C-reactive protein level of 31.8 mg/L, and alkaline phosphatase was markedly elevated at 2987 IU/L (Table 2). Imaging of the hips and knees was unremarkable. On physical examination, the patient demonstrated significant hepatosplenomegaly and abdominal distension without lymphadenopathy. Given these findings, the differential diagnosis included hematologic malignancy, viral or fungal infection, an atypical autoimmune process, Paget’s disease, and multiple myeloma.

**Table 1:** Complete Blood Count With Differential During ED Workup ED, emergency department.

Parameter (Units)	ED Workup	Reference Range
White blood cells (k/µL)	46.69	6.0-17.5
Red blood cells (M/µL)	3.84	3.50-5.00
Hemoglobin (g/dL)	10.0	11.2-14.3
Hematocrit (%)	31.1	34.0-40.0
Mean corpuscular volume (fL)	80.9	75.0-87.0
Mean corpuscular hemoglobin (pg)	26.0	23.0-31.0
Mean corpuscular hemoglobin concentration (g/dL)	32.2	30.0-37.0
Red cell distribution width (%)	14.8	11.5-15.5
Platelet count (k/µL)	166	150-450
Neutrophils (%)	8.0	15.0-35.0
Lymphocytes (%)	83.0	41.0-71.0
Monocytes (%)	5.0	3.0-13.0
Eosinophils (%)	3.0	0.0-7.0
Basophils (%)	1.0	<2.0
Neutrophils, absolute (k/µL)	3.20	1.5-8.0
Lymphocytes, absolute (k/µL)	38.75	3.0-10.5
Monocytes, absolute (k/µL)	2.33	0.2-1.2
Eosinophils, absolute (k/µL)	1.40	0.0-0.6
Basophil, absolute (k/µL)	0.47	0.0-0.1

**Table 2:** Alkaline Phosphatase and C-Reactive Protein During ED Workup ED, emergency department.

Parameter (Units)	ED Workup	Reference Range
Alkaline phosphatase (IU/L)	2987	140-335
C-reactive protein (mg/L)	31.8	5-10

**Figure 1:**
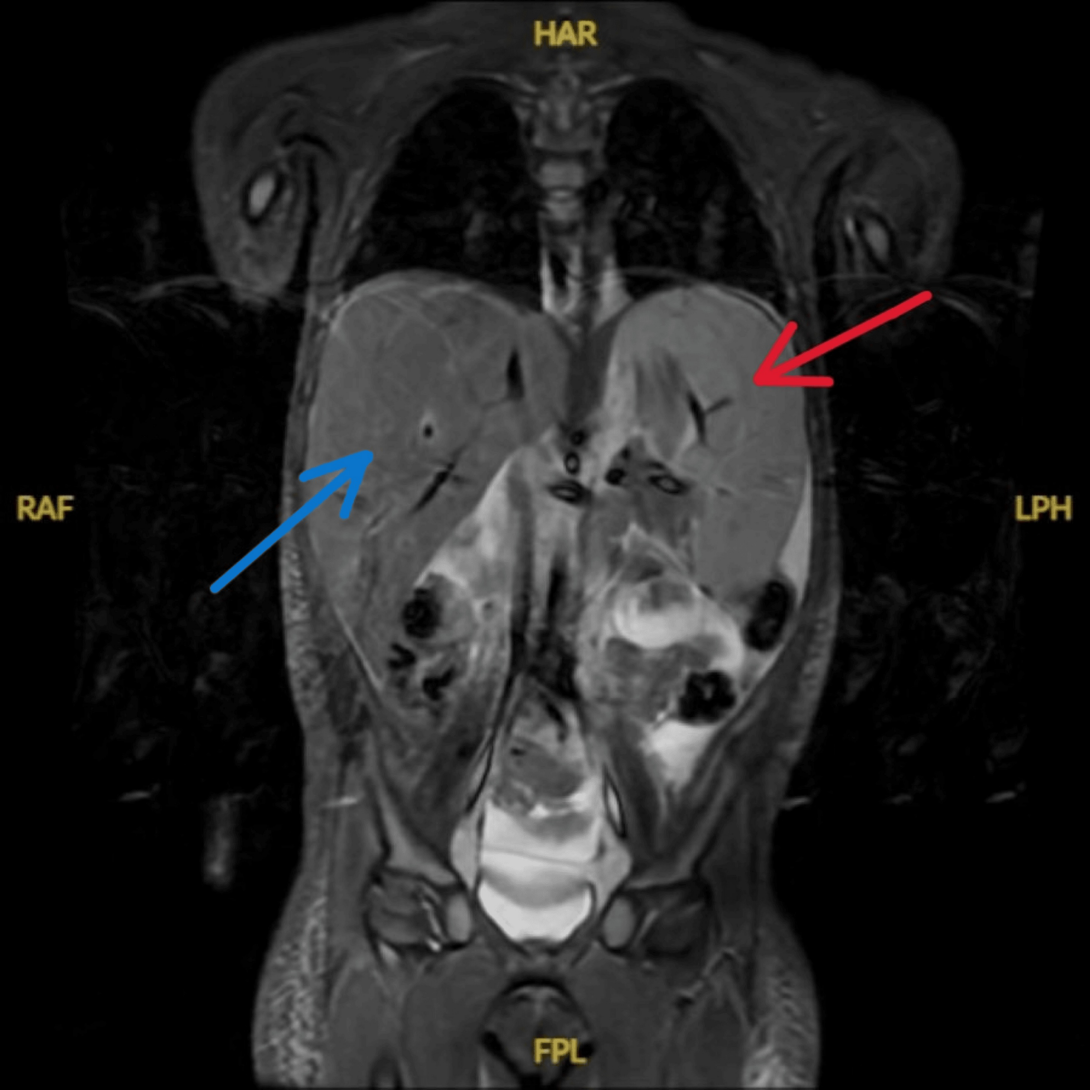
Whole-Body MRI (Coronal View) Demonstrating Massive Hepatomegaly (Blue Arrow) and Splenomegaly (Red Arrow) MRI, magnetic resonance imaging.

**Figure 2:**
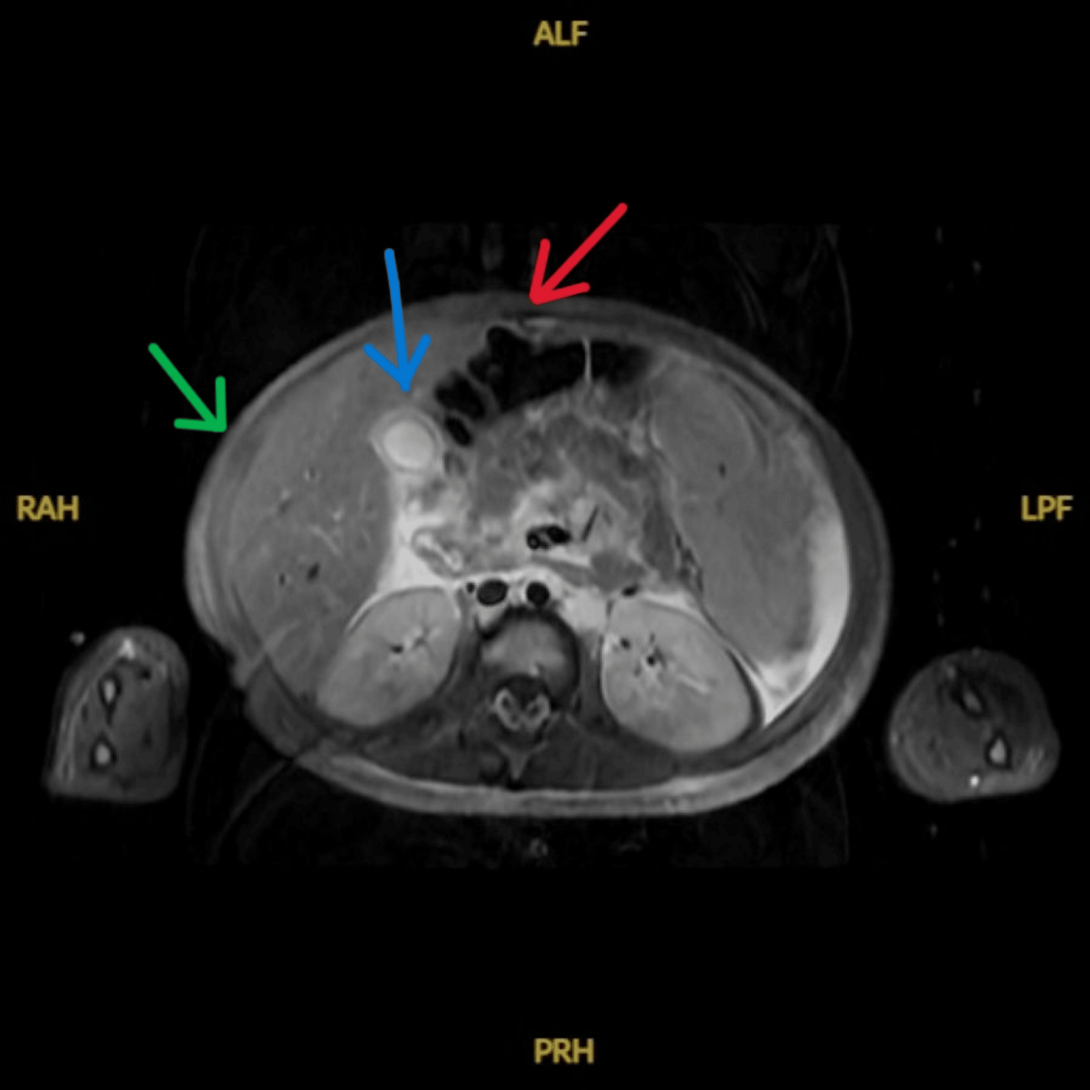
Whole-Body MRI (Axial View) Demonstrating Diffuse Anasarca (Green Arrow), Ascites (Red Arrow), and Gallbladder Edema (Blue Arrow) MRI, magnetic resonance imaging.

Four days after ED workup, the patient was admitted to the hematology/oncology service. Peripheral blood smear demonstrated lymphocytosis with atypical lymphocytes. Large granular lymphocytes with irregular nuclear contours and clumped chromatin without obvious nucleoli or blasts were observed. Bone marrow biopsy revealed a hypercellularity of 90%, with 40% comprising γδ T-cells. Flow cytometry of the bone marrow revealed T-cells comprising 97% of lymphoid-gated events and 63% of total cells, expressing CD45, CD2, CD7, extensive CD56, and dim CD5, while lacking CD4, CD8, and TRBC1 expression. Conventional cytogenetic analysis revealed a normal female karyotype, and the standard acute lymphoblastic leukemia (ALL) fluorescence in situ hybridization (FISH) panel was negative for common ALL-associated abnormalities. Comprehensive molecular profiling specific to HSTCL was not performed. Whole-body coronal magnetic resonance imaging (MRI) showed massive hepatosplenomegaly, with the spleen measured to be 13.4 cm (Figure 1). Axial MRI revealed significant anasarca and diffuse homogeneous marrow signal abnormality, consistent with extensive marrow infiltration (Figure 2). Diffuse gallbladder wall edema, moderate ascites, and abdominal subcutaneous body wall edema were also present (Figure 2). Repeat CBC showed declines in red blood cell count, hematocrit, hemoglobin, and platelets compared to the ED evaluation. Flow cytometry of a liver biopsy revealed 97% γδ T-cells. A repeat bone marrow biopsy, consistent with prior findings, confirmed the diagnosis of γδ HSTCL.

The patient initiated chemotherapy according to a pediatric T-cell protocol (AALL1231/0434) but exhibited refractory disease following standard induction therapy. On day 15 of treatment, therapy was escalated per protocol to consolidative regimens including nelarabine, cyclophosphamide, cytarabine, and 6-mercaptopurine. Despite these interventions, remission was not achieved. Post-consolidation bone marrow evaluation demonstrated a small abnormal T-cell population on flow cytometry, confirming MRD (minimal residual disease) positivity. MRD negativity is defined as <0.01% of nucleated mononuclear cells. Four weeks of high-dose methotrexate reduced residual disease to 3-4% of nuclear cells. Given persistent MRD positivity and refractory disease, therapy was subsequently switched to daratumumab, venetoclax, and azacitidine. This combination was selected based on biologic considerations in the setting of refractory disease, with venetoclax and azacitidine chosen to target apoptotic pathway dependence and daratumumab added for its immunomodulatory effects, including depletion of CD38+ immune cells and potential enhancement of anti-tumor immune responses. ClonoSEQ testing two months into this regimen demonstrated a partial decrease in clonal burden from 8,112 to 4,908 copies. Flow cytometry at Children's Hospital Los Angeles (CHLA) detected 0.68% abnormal nucleated mononuclear cells, confirming persistent MRD positivity. Due to persistent MRD positivity, the patient was deemed ineligible for allogeneic stem cell transplantation at that time, as MRD negativity is required to optimize transplant outcomes and minimize the risk of relapse. Treatment with daratumumab, venetoclax, and azacitidine will continue until MRD negativity is achieved, at which point she will proceed to planned haploidentical stem cell transplantation from her father.

The patient’s treatment course was complicated by febrile neutropenia, multiple episodes of multi-drug-resistant *Staphylococcus epidermidis* and *Escherichia coli* bacteremia, and cytomegalovirus viremia. She also developed worsening anasarca, steroid-induced hypertension, and poor oral intake necessitating gastrostomy tube placement. Additionally, she experienced an episode of periumbilical cellulitis. Her constipation persisted and was managed with polyethylene glycol.

## Discussion

γδ HSTCL is an exceptionally rare and highly aggressive peripheral T-cell neoplasm, particularly in pediatric populations. Its rarity and nonspecific presentation frequently result in delayed diagnosis. This case highlights these challenges: an 18-month-old initially evaluated for benign-appearing gastrointestinal symptoms, constipation, and intermittent hematochezia, who later developed prolonged fevers, progressive weight loss, hepatosplenomegaly, and evolving cytopenias. Early manifestations of γδ HSTCL can be subtle or misleading, and the absence of lymphadenopathy often directs physicians toward more common pediatric conditions before malignancy is considered [7]. 

Accurate diagnosis in this case was ultimately achieved through immunophenotypic, molecular, and histopathologic evaluation. The patient’s immunophenotype with expression of CD45, CD2, CD7, and CD56 with dim CD5, and absence of CD4, CD8, and TRBC1, was consistent with classical γδ T-cell lineage. These findings, along with marked hepatosplenomegaly and diffuse marrow and hepatic infiltration, align with adult presentations of γδ HSTCL, as described in the literature [7]. However, pediatric γδ HSTCL remains extraordinarily uncommon, and this case underscores the importance of maintaining clinical suspicion when persistent systemic symptoms accompany cytopenias and organomegaly.

Characteristic genetic features of HSTCL include isochromosome 7q, trisomy 8, and activating mutations of JAK/STAT pathway genes (*STAT5B*, *STAT3*) and chromatin-modifying genes (*SETD2*, *PTEN*, *INO80*, *ARID1B*) [8]. In this patient, conventional cytogenetic analysis revealed a normal female karyotype, and the standard ALL FISH panel was negative for common ALL-associated abnormalities. The absence of isochromosome 7q and trisomy 8, which are present in approximately 42% and 33% of HSTCL cases, respectively, represents an atypical cytogenetic presentation [8]. National Comprehensive Cancer Network (NCCN) guidelines recommend conventional chromosome analysis and FISH for isochromosome 7q and trisomy 8 as useful diagnostic tests in HSTCL [8]. Multigene panel testing for *STAT3*, *STAT5B*, *SETD2*, and *INO80* mutations may provide additional diagnostic and prognostic information [8]. Comprehensive molecular profiling beyond standard ALL panels was not performed in this case, representing a limitation in fully characterizing the molecular landscape of this patient's disease. These genetic alterations are increasingly recognized as part of HSTCL pathogenesis and may represent potential therapeutic targets.

Treatment of γδ HSTCL in children is particularly challenging due to its aggressive biology, poor response to conventional chemotherapy, and the absence of well-established pediatric treatment guidelines. This patient demonstrated refractory disease despite intensive induction and consolidation treatment regimens. HSTCL exhibits marked chemoresistance to anthracycline-based regimens, with complete remission being extremely uncommon and most patients dying from lymphoma within two years of diagnosis [9]. Recent data suggest that non-anthracycline regimens, particularly platinum-based therapies such as ICE (ifosfamide, carboplatin, etoposide) or IVAC (ifosfamide, etoposide, high-dose cytarabine), may have better outcomes for remission [9]. In a retrospective analysis of 20 patients, non-anthracycline regimens showed higher response rates (83% vs. 50%) compared to anthracycline-based therapy, with a trend toward improved three-year overall survival (100% vs. 29%) [9].

Molecular and flow cytometric monitoring of MRD played a pivotal role in assessing therapeutic response. Persistent MRD positivity and incomplete reduction of clonal burden on ClonoSEQ analysis emphasize the characteristic chemotherapy resistance of γδ HSTCL and the need for early planning toward hematopoietic stem cell transplantation [10]. Achieving MRD negativity (<0.01%) remains a critical benchmark before proceeding with transplant. High-throughput sequencing of TCR genes has become a sensitive method for MRD detection in lymphomas and can guide therapeutic decisions [10].

This patient’s clinical course was further complicated by substantial treatment-related toxicities, including recurrent febrile neutropenia, multidrug-resistant bacteremia, cytomegalovirus viremia, steroid-induced hypertension, worsening anasarca, and nutritional compromise. These complications reflect not only the intensity and toxicity of current regimens but also the need for comprehensive multidisciplinary supportive care.

Despite the aggressive nature of her disease and the complexity of her clinical course, the patient has shown encouraging response trends, including partial reduction in clonal burden and stabilization of systemic symptoms. Continued chemotherapy with the goal of achieving MRD negativity for transplant eligibility offers a path toward potential long-term remission. Long-term remission in HSTCL is primarily seen in those who have undergone consolidative hematopoietic cell transplantation (HCT) [11]. Multiple studies confirm that allogeneic HCT offers the only definitive cure for HSTCL [11]. In the largest Mayo Clinic series, patients who underwent HCT had significantly higher three-year overall survival compared to non-recipients (83% vs. 33%). The European Society for Blood and Marrow Transplantation recommends that patients who respond to first-line treatment and are eligible for transplantation should be offered allogeneic HCT whenever possible [12].

Overall, this case illustrates the diagnostic difficulty, therapeutic complexity, and clinical urgency associated with pediatric γδ HSTCL. Early recognition, prompt initiation of appropriate diagnostic studies, such as immunophenotyping and molecular MRD assessment, and timely escalation to intensive therapy are critical to improving outcomes.

## Conclusions

This case demonstrates the extreme rarity and aggressive clinical course of pediatric γδ HSTCL, a malignancy that is often difficult to recognize early due to its nonspecific symptoms and lack of lymphadenopathy. In very young children, γδ HSTCL should be considered early when hepatosplenomegaly and cytopenias coexist, as delayed recognition may limit timely access to curative therapy. The patient’s persistent MRD, despite multiple intensive chemotherapy regimens, highlights the refractory nature of γδ HSTCL and reinforces the essential role of highly sensitive MRD surveillance, using flow cytometry and molecular assays, to guide therapeutic decision-making and enable curative hematopoietic stem cell transplantation. Emerging genomic findings, including *PTEN* and *SETD2* alterations, may provide future insights into disease pathogenesis, although their prognostic significance in pediatric HSTCL is yet to be defined. Ultimately, this case emphasizes that improved outcomes rely on early disease recognition, prompt comprehensive immunophenotypic and molecular evaluation, and timely pursuit of hematopoietic stem cell transplantation. Continued reporting of pediatric γδ HSTCL cases is critical to advance understanding of the disease, refine diagnostic algorithms, optimize treatment strategies, and improve prognoses for children affected by this challenging disease.
